# Biomarkers Predictive of Extubation and Survival of COVID-19 Patients

**DOI:** 10.7759/cureus.15462

**Published:** 2021-06-05

**Authors:** Gregory Topp, Megan Bouyea, Nicholas Cochran-Caggiano, Ashar Ata, Pedro Torres, Jackcy Jacob, Danielle Wales

**Affiliations:** 1 Department of Medicine, Albany Medical College, Albany, USA; 2 Department of Surgery, Albany Medical Center, Albany, USA; 3 Department of Medicine, Albany Medical Center, Albany, USA; 4 School of Public Health, State University of New York at Albany, Albany, USA

**Keywords:** covid 19, biomarkers, intubation, extubation, platelet count

## Abstract

Purpose

Many patients with COVID-19 who develop acute respiratory distress syndrome (ARDS) require prolonged periods of mechanical ventilation. Mechanical ventilation may amplify ventilator-associated complications and extend resource utilization. A better understanding of prognostic indicators could help in the planning and distribution of resources, particularly in resource-limited areas. We analyzed laboratory studies of intubated COVID-19 patients with the goal of identifying biomarkers that may predict extubation success and survival to discharge.

Methods

A retrospective chart review was performed on all COVID-19 patients requiring mechanical ventilation between January 3, 2020, and January 7, 2020, in a single academic tertiary care center in Northeastern New York State. The electronic medical record was used to collect 14 laboratory variables at three time points: admission, intubation, and extubation (including terminal extubation) for all intubated intensive care unit (ICU) patients treated for COVID-19. Mean laboratory values were analyzed with the Mann-Whitney U test. Categorical variables were analyzed with the two-sample Wilcoxon rank-sum test.

Results

Seventy-two patients met the inclusion criteria. Forty-three patients were male. The mean age was 61 years. The overall mortality was 50%. On admission, intubated patients who survived had significantly higher platelet counts (p=0.024), and absolute lymphocyte counts (ALC; p=0.047). Notably, ferritin (p=0.018) and aspartate transaminase (AST; p=0.0045) levels were lower in survivors.

At the time of intubation, survivors again had a higher platelet count (p=0.024) and ALC (p=0.037) levels. They had a lower D-dimer (p=0.0014), ferritin (p=0.0015), lactate dehydrogenase (LDH; p=0.0145), and AST (p=0.018) compared to intubated patients who died.

At extubation, survivors had higher platelet count (p=0.0002), ALC (p=0.0013), and neutrophil/lymphocyte ratio (NLR; p=0.0024). Survivors had lower d-dimer (p=0.035), ferritin (p=0.0012), CRP (p=0.045), LDH (p=0.002), AST (p<0.001), and ALK (p=0.0048).

Conclusions

Biomarkers associated with increased risk of mortality include platelet count, ALC, lymphocyte percentage, NLR, D-dimer, ferritin, C-reactive protein (CRP), AST, alanine transaminase (ALT), and alkaline phosphatase (ALK). This study provides additional evidence that these biomarkers have prognostic value in patients with severe COVID-19. The goal is to find objective surrogate markers of disease improvement or success of extubation. When considered within the larger body of data, it is our hope that a mortality risk calculator can be generated for intubated COVID-19 patients.

## Introduction

In 2019, novel coronavirus SARS-CoV-2 emerged as a major pathogen. Causing the illness known as COVID-19, it became a major cause of morbidity and mortality across the globe. The primary clinical presentations of infection include fever, cough, and shortness of breath, with the potential to progress to severe acute respiratory distress syndrome (ARDS) and cytokine storm [1]. Inflammatory markers have been found to correlate with the severity of disease presentation but have not been analyzed for their predictive value [2]. Pneumocytes with viral cytopathic effect have been discovered, which implies direct viral damage to these cells [3].

A significant number of patients infected with COVID-19 require mechanical ventilation [4]. In a case series from the Seattle area, 75% of patients admitted with hypoxic respiratory failure and COVID-19 required mechanical ventilation, for a mean duration of approximately 10 days among the survivors [5]. Albeit lifesaving in many cases, mechanical ventilation poses many risks, including lung injury, pneumothorax, ventilator-associated pneumonia, and adverse effects from sedatives and paralytics [6]. Therefore, it is critical to minimize the time a patient is on mechanical ventilatory support. Supplementing clinical with laboratory data to identify patients who will require longer periods of mechanical intubation is essential. This patient population is at high risk of unsuccessful extubation as well as complications of prolonged intubation and mechanical ventilation, including death. This supplemental information could aid in resource allocation and planning in resource-limited environments and situations, such as a pandemic. While there have been studies documenting trends in clinical biomarkers in relation to disease severity, to date, there has not been an evaluation of clinical biomarkers at the time of initiation and cessation of mechanical ventilation [1,7,8].

This study analyzes laboratory studies in patients with COVID-19 to determine if certain laboratory values are predictive of survival after extubation.

## Materials and methods

All intubated patients diagnosed with COVID-19 admitted to Albany Medical Center (AMC) in Albany, New York between March 1, 2020 and July 1, 2020 were identified by billing records and the electronic health record (Soarian Clinicals®). Records were subsequently de-identified. Data collection was retrospective and included patient demographics, labs, and final disposition (i.e., death, survival, and discharge from the hospital). Inclusion criteria included adult patients (age 18 and above) who required mechanical ventilation in an ICU setting and a positive COVID-19 RT-PCR via nasal swab or endotracheal sampling. Successful extubation was defined as a patient being extubated and discharged alive from the hospital. Many patients were extubated to comfort care and eventual death. This terminal extubation was considered a death. Patients who opted for DNI (Do Not Intubate) were excluded from the analysis. This protocol was reviewed by the Albany Medical College Institutional Review Board (protocol #5873) and determined to be exempt.

Once patients were identified, pertinent variables such as date of admission, intubation, and extubation (including terminal extubation as well as patients who survived extubation) were obtained from Soarian. Patients' charts were reviewed to see if they received any treatments (e.g., hydroxychloroquine, azithromycin, etc.) during their hospitalization, as well as prior to their transfer to our center, if applicable. Laboratory studies from the inpatient stay and documented comorbidities including COPD, diabetes, heart disease, renal failure, and cirrhosis were analyzed. Laboratory values were collected on the date of admission, date of intubation, and date of extubation. If laboratory values were not available on the dates of those events, the most recent laboratory values were used. These laboratory values (with abbreviations noted in parentheses where appropriate) include white blood cell count (WBC), hemoglobin (Hb), hematocrit (Hct), platelet count, lymphocyte percent, neutrophil: lymphocyte ratio (NLR), absolute lymphocyte count (ALC), D-dimer, ferritin, C-reactive protein (CRP), lactate dehydrogenase (LDH), aspartate aminotransferase (AST), alanine transaminase (ALT), and alkaline phosphatase (ALK). Ferritin was analyzed by sex, as males and females have different normal values at baseline. Hematologic values (i.e., WBC, Hb, Hct, platelets, lymphocyte percent, NLR, ALC) were obtained using the Sysmex XN-9000™ (Sysmex Co., Kobe, Japan). The D-dimer assay was from Instrumentation Laboratory®, Bedford, MA, USA. AST, ALT, ALK, CRP, and LDH were obtained via an assay from the AU series (Beckman Coulter Life Sciences®, Indianapolis, IN, USA). Ferritin assay was DxI (Beckman Coulter Life Sciences®). Demographic values were analyzed using Fisher's exact t-test. Laboratory values were analyzed using a Mann-Whitney U test. Fisher's exact t-test was used in the case of ALT, as the data were noted to be significantly skewed. Comorbidities were compared using Poisson regression.

## Results

Data analysis was conducted on a total of 72 patients who met inclusion criteria. Thirty-eight of those patients were transferred to our facility. Forty-three (59.7%) patients were male, and 29 (40.2%) patients were female. The mean age at the time of hospitalization was 60.75 ± 15.18 (mean ± SD) with a range of 22-90 years. Twenty-seven (37.5%) patients were white, 17 (23.6%) patients were black, 7 (9.7%) patients were Asian, and 21 (29.2%) patients were of unknown race. Fifty-eight (80.5%) patients were non-Hispanic. Of the 72 patients included, 36 (50%) patients were successfully extubated, while 36 (50%) patients died due to complications from COVID-19. Sixteen of the deaths were from patients transferred to our facility. One patient did require reintubation and survived. Demographic data are summarized in Tables 1 and 2, while Tables 3 and 4 summarize relevant comorbidities and treatments. Although extracorporeal membrane oxygenation (ECMO) and thiamine were significant predictors of mortality, it should be noted that the sample size for both treatments was very small at 2.

**Table 1:** General patient demographics (n=72).

Demographic variable	Value
Mean age ± SD	60.75 ± 15.18
Age range	22–90
Male sex	43 (59.7%)
Female sex	29 (40.2%)
Hispanic ethnicity	12 (16.7%)
Non-Hispanic ethnicity	60 (83.3%)
White race	30 (41.7%)
Black race	16 (22.2%)
Asian race	8 (11.1%)
Unknown race	18 (25%)

**Table 2:** Demographics of patients successfully extubated versus patients who died. *Statistical significance.

Demographic variable	Survived (n=36)	Death (n=36)	p-Value
Mean age ± SD	63.61 ±16.7	57.88±13.15	0.0419*
Age range	22–80	31–90	
Male sex	22 (53.6%)	19 (46.3%)	
Female sex	14 (45.2%)	17 (54.8%)	0.475
White race	13 (36.1%)	17 (47.2%)	
Black race	8 (22.2%)	8 (22.2%)	
Asian race	6 (16.7%)	2 (5.6%)	
Unknown race	9 (25%)	9 (25%)	0.496

**Table 3:** Unadjusted bivariate effects (incident risk ratios) of comorbidities on the risk of mortality among intubated COVID patients. *Statistical significance. IRR: incident risk ratio, COPD: chronic obstructive pulmonary disease.

Comorbidity	IRR	Confidence interval
COPD	1.32	0.78–2.22
Hypertension	0.73	0.45–1.19
Coronary artery disease	0.86	0.40–1.89
Diabetes	0.72	0.43–1.21
Chronic kidney disease	1.58	0.98–2.55
Sepsis	1.31	0.81–2.13
Shock	1.92	1.18–3.13*
Acute renal failure	1.23	0.76–2.01
Cirrhosis	2.36	1.75–3.19*

**Table 4:** Unadjusted bivariate effects (incident risk ratios) of treatment type on the risk of mortality among intubated COVID patients. *Statistical significance. IRR: incident risk ratio, ECMO: extracorporeal membrane oxygenation.

Treatment	IRR	Confidence interval
Hydroxychloroquine	0.81	0.51-1.29
Azithromycin	0.66	0.42-1.03
Remdesevir	0.46	0.82-2.59
Convalescent plasma	0.50	0.24-1.02
Vitamin C	0.62	0.12-3.18
Thiamine	1.97	1.52-2.54*
Zinc	0.95	0.23-3.93
ECMO	1.64	1.12-2.34*
Steroids	0.56	0.26-1.23

Laboratory values were collected for each patient at the time of admission to the ICU, at the time of mechanical intubation, and at the time of extubation or death. Collected laboratory data (Table 5) included WBC, Hb, Hct, platelet count, lymphocyte percent, NLR, ALC, D-dimer, ferritin, CRP, LDH, AST, ALT, and ALK.

**Table 5:** Mean laboratory values of COVID-19 ICU patients at the time of admission, intubation, and extubation. *Statistical significance. WBC: white blood count, CRP: C-reactive protein, LDH: lactate dehydrogenase, AST: aspartate aminotransferase, ALT: alanine aminotransferase; ALK: alkaline phosphatase, FEU: fibrinogen equivalent units.

	Admission			Intubation			Extubation		
Laboratory Test	Survival	Death	P-value	Survival	Death	P-value	Survival	Death	P-value
WBC (103/µL)	11.40	9.87	0.12	11.78	11.53	0.51	12.3	16.96	0.16
Hemoglobin (g/dL)	11.88	11.95	0.85	11.394	11.94	0.57	9.82	9.57	0.69
Hematocrit (%)	37.38	37.79	0.97	35.97	37.24	0.69	31.67	30.67	0.43
Platelet (103/µL)	257.73	215.60	0.024*	282.18	221.85	0.024*	327.71	203.78	0.0002*
Lymphocyte %	10.065	8.30	0.58	9.063	6.71	0.11	12.15	7.7	0.0013*
Neutrophil/lymphocyte ratio	16.20	15.23	0.55	16.29	23.58	0.15	10.57	25.94	0.0024*
Absolute lymphocyte	1298.78	693.54	0.047*	1037.22	709.85	0.037*	1324.61	909.43	0.0096*
D-dimer (mg/L FEU)	18.82	24.49	0.21	10.40	83.45	0.0014*	3.60	51.089	0.035*
Ferritin - total (ng/ml)	550.23	1056.65	0.018*	687.70	1910	0.0015*	613.83	2793.68	0.0012*
Ferritin - male (ng/ml)	901	976.31	0.064	994.4	2058.4	0.0597	809.15	2948.42	0.0094*
Ferritin - female (ng/ml)	257.92	1205.86	0.0127*	304.33	1662.67	0.0033*	359.9	2528.43	0.0231*
CRP (mg/L)	190.83	160.077	0.67	186.24	186.046	0.84	84.1	140.01	0.0455*
LDH (IU/L)	392.046	488.94	0.058	398.88	663.52	0.0145*	305.28	1473.42	0.0020*
AST (IU/L)	44.064	81.7	0.0045*	43.91	334.76	0.018*	34.35	1019.57	<0.001*
ALT (IU/L)	43.031	50.22	0.24	42.031	83	0.29	50.18	451.19	0.1618
ALK (IU/L)	75.47	115.03	0.099	79.28	127	0.089	78.35	159.53	0.0048*

Laboratory results

On admission, patients who survived had significantly higher platelet count (p=0.024) and ALC (p=0.047), while AST (p=0.0045) and ferritin in female patients (p=0.0127) were significantly lower.

At the time of intubation, survivors had higher platelet count (p=0.024) and ALC (p=0.037). Survivors also had a significantly lower d-dimer (p=0.0014), LDH (p=0.0145), and AST (p=0.018) at this time point. Ferritin was again significantly lower in female patients.

On extubation or death, survivors had higher platelet count (p=0.0002), lymphocyte percent (p=0.0013), and NLR (p=0.0024). Survivors had lower D-dimer (p=0.035), CRP (p=0.045), LDH (p=0.002), AST (p<0.001), and ALK (p=0.0048). Ferritin was significantly lower in both sexes.

## Discussion

With the novel coronavirus SARS-CoV-2 infecting over 29 million in the United States as of March 2021, a massive burden has been placed on the United States healthcare system [9]. There is a dire need to identify high-risk COVID-19 patients who need mechanical ventilation support and subsequently predict patient outcomes to guide clinical management. Laboratory biomarkers have the potential to provide valuable prognostic information for patients diagnosed with COVID-19 infection.

It should be noted that the age of patients who died was significantly younger than those who extubated and survived. This may indicate that younger patients were more likely to receive aggressive interventions, including ECMO and mechanical ventilation. Older patients may not have been intubated at the same rate, selecting outpatients with more comorbidities and increased risk of death on a ventilator. This may also indicate greater advance care planning among older patients, or discussion with the medical team regarding goals of care prior to intubation.

Known complications of severe COVID-19 infection include mild thrombocytopenia and hypercoagulable state, which lead to the formation of micro-and macrothrombi [10]. These complications may precede disseminated intravascular coagulation or multiorgan failure. Our finding of decreased platelet count in patients who succumbed to severe COVID-19 infection is consistent with previous studies, which provide evidence of a relationship between thrombocytopenia and disease severity [5]. In intubated patients who did not survive COVID-19 infection, D-dimer was significantly elevated at intubation and extubation in those who died. Literature indicates that elevated D-dimer may reflect thrombogenesis or inflammatory dysregulation and is associated with increased disease severity [1,11]. Both decreased platelet count and D-dimer elevation are associated with poor patient outcomes and support systemic microthrombus formation.

A pro-inflammatory state and inflammatory dysregulation are also associated with COVID-19 infection leading to a so-called “cytokine storm.” Our finding of hyperferritinemia, particularly in female patients, is therefore expected secondary to inflammation and is associated with an increased risk of death. The association between hyperferritinemia and disease severity has also been reported by other researchers [12]. Similarly, elevations in CRP and LDH are also associated with poor outcomes.

A relative lymphocytopenia was present upon admission, intubation, and extubation in patients who did not survive COVID-19 infection. This finding resembles previous studies, which provided evidence of a relationship between low lymphocyte counts and both increased infection severity and mortality [13]. The pathogenesis of relative lymphocytopenia in COVID-19 remains unknown. However, hypotheses include vascular sequestration and apoptosis, such as in SARS, or susceptibility of lymphocytes to viral attack due to ACE2 receptor expression [10,14].

Of importance are the significant findings of higher lymphocyte percentage, ALC, and NLR as predictors of survival. Based on our results, we hypothesize that a higher lymphocyte percentage and lymphocyte count may be associated with lower physiologic stress, and therefore, patients may be more likely to survive extubation. Furthermore, these findings may be the result of steroid administration, which became more common during this period. Systemic corticosteroids are known to increase circulating neutrophil counts and decrease lymphocyte counts. Dexamethasone was noted to be the most common corticosteroid used during the time of this study.

Abnormalities in transaminases upon hospital admission have been previously linked to increased severity of COVID-19 infection [11]. In this study, we have further characterized transaminase abnormalities throughout the duration of illness. Although ALT elevation was noted at extubation in patients who did not survive COVID-19 infection, this data point was not found to be significant, based on the Mann-Whitney U test. It should be noted that there was a large standard deviation (SD=126.3) at extubation which likely affects results. This finding is in part due to two significant outliers who did not survive. In fact, re-analysis with a one-sided Fisher’s exact t-test shows a p-value at the admission of 0.074, whereas the p-value at intubation was 0.036, and at extubation was 0.043. This indicates that ALT at intubation and extubation was significantly different. This is a similar finding to a large retrospective study from New York City where levels of AST were notably higher than levels of ALT [15]. Further research comparing AST and ALT at extubation is indicated to explore this finding. One possible explanation is that the patients suffered from autoimmune hemolytic anemia which has been previously described as a possible complication of COVID-19 [16]. Our findings are not consistent with an intravascular hemolysis hypothesis, but further study is warranted. Another possibility is that elevated transaminases, particularly ALT, may be associated with hepatic congestion due to right heart dysfunction in the setting of high pulmonary pressures seen in intubated ARDS patients. It is also possible that the AST may be elevated not only due to liver dysfunction but also to non-hepatic causes.

Limitations include the use of data sourced from a single institution and potential confounders due to the retrospective nature of data collection. Potential unmeasured confounders include volume status, nutritional status, and concurrent infection. Furthermore, patients were not excluded from this study based on pre-existing health conditions, such as chronic liver disease. There may be other laboratory studies that play an important role in the prognosis of intubated COVID-19 patients. Most laboratory studies were conducted at our institution; however, several patients were transferred from outside hospitals. Some results from external hospitals were not available, therefore labs upon initial presentation to Albany Medical Center were utilized as the admission time point. This may affect the comparison between labs at admission compared to intubation and extubation. These factors in combination with this study’s small sample size warrant further investigation using a larger dataset.

In summary, laboratory biomarkers have the potential to provide valuable prognostic indications for patients diagnosed with severe COVID-19 infection. This study reveals distinct trends in inflammatory markers and LFTs associated with poor outcomes among COVID-19 patients requiring mechanical ventilation. In the future, acute phase reactants and biomarkers including but not limited to platelet count, ALC, ferritin, and AST/ALT have the potential to be used for risk stratification of COVID-19 patients. Objective laboratory values could provide a framework for determining the likelihood of disease improvement, the success of extubation, and aid with resource allocation. Our hope is that these data will be used in a comprehensive meta-analysis, or to generate a mortality risk calculator for intubated COVID-19 patients. This tool could assist in guiding clinical management and provide important prognostic information for the clinical team, family members, and health care proxies who may need to make end-of-life decisions.

## Conclusions

Upon hospital admission, platelet count, ALC, ferritin (for female patients), and AST levels were significantly higher in mechanically ventilated COVID-19 patients who did not survive. As the disease progressed, D-dimer and LDH levels became significantly elevated at the point of intubation. At extubation, lymphocyte %, NLR, ferritin (for both sexes), and CRP were also significantly different between groups. This study monitored laboratory values throughout the duration of illness and provides the initial framework for utilizing biomarkers to predict the prognosis of intubated COVID-19 patients.
